# Current T_1_ and T_2_ mapping techniques applied with simple thresholds cannot discriminate acute from chronic myocadial infarction on an individual patient basis: a pilot study

**DOI:** 10.1186/s12880-016-0135-y

**Published:** 2016-04-29

**Authors:** Florian von Knobelsdorff-Brenkenhoff, Marcel Prothmann, Matthias A. Dieringer, Ralf Wassmuth, André Rudolph, Wolfgang Utz, Julius Traber, Andreas Greiser, Thoralf Niendorf, Jeanette Schulz-Menger

**Affiliations:** Working Group Cardiovascular Magnetic Resonance, Experimental and Clinical Research Center, a joint cooperation between the Charité Medical Faculty and the Max-Delbrueck Center for Molecular Medicine and HELIOS Klinikum Berlin Buch, Department of Cardiology and Nephrology, Lindenberger Weg 80, Berlin, 13125 Germany; Berlin Ultrahigh Field Facility, Max-Delbrueck Center for Molecular Medicine, Robert-Rössle-Str. 10, Berlin, 13125 Germany; Siemens Healthcare, Allee am Roethelheimpark 2, Erlangen, 91052 Germany; Experimental and Clinical Research Center, a joint cooperation between the Charité Medical Faculty and the Max-Delbrueck Center for Molecular Medicine, Lindenberger Weg 80, Berlin, 13125 Germany

**Keywords:** Magnetic resonance, Myocardial infarction, Mapping

## Abstract

**Background:**

Studying T_1_- and T_2_-mapping for discrimination of acute from chronic myocardial infarction (AMI, CMI).

**Methods:**

Eight patients with AMI underwent CMR at 3 T acutely and after >3 months. Imaging techniques included: T_2_-weighted imaging, late enhancement (LGE), T_2_-mapping, native and post-contrast T_1_-mapping. Myocardial T_2_- and T_1_-relaxation times were determined for every voxel. Abnormal voxels as defined by having T_2_- and T_1_-values beyond a predefined threshold (T_2_ > 50 ms, native T_1_ > 1250 ms and post-contrast T_1_ < 350 ms) were highlighted and compared with LGE as the reference.

**Results:**

Abnormal T_2_-relaxation times were present in the voxels with AMI (=> delete acute infarction; unfortunately this is not possible in your web interface) acute infarction only in half of the subjects. Abnormal T_2_-values were also present in subjects with CMI, thereby matching the chronically infarcted territory in some. Abnormal native T_1_ times were present in voxels with AMI in 5/8 subjects, but also remote from the infarcted territory in four. In CMI, abnormal native T_1_ values corresponded with infarcted voxels, but were also abnormal remote from the infarcted territory. Voxels with abnormal post-contrast T_1_-relaxation times agreed well with LGE in AMI and CMI.

**Conclusions:**

In this pilot-study, T_2_- and T_1_-mapping with simple thresholds did not facilitate the discrimination of AMI and CMI.

## Background

Cardiovascular magnetic resonance enables myocardial tissue characterization by combining native and contrast-enhanced techniques with differences in T_2_- and T_1_-weighting. Native T_2_-weighted imaging has been reported to detect myocardial edema [1, 2], and T_1_-weighted late Gadolinium enhancement imaging (LGE) has been established to show necrosis and fibrosis. Recent studies demonstrated that the use of these techniques allows the differentiation of acute from chronic myocardial infarction (AMI, CMI) [3]. However, there is an ongoing controversial debate about the technical limitations and the pathophysiologic background of conventional T_2_-weighted edema imaging [4, 5]. Recently, myocardial T_1_- and T_2_-mapping were introduced to quantify the T_1_- and T_2_-relaxation times, which may be superior to the semiquantitative or qualitative image assessment used with conventional T_2_-weighted imaging [6–8]. For patients with AMI, prolonged native T_2_- and T_1_-relaxation times as well as decreased post-contrast T_1_-relaxation times were reported for the infarcted areas [9–12]. In patients with CMI, increased native and decreased post-contrast T_1_-relaxation times were reported [12]. However, whether the utilization of T_2_- and T_1_-mapping helps to differentiate AMI and CMI in the individual patient has not been examined in detail. We hypothesized that applying T_1_- and T_2_-mapping with simple thresholds based on reference values from healthy controls will discriminate AMI and CMI on an individual patient basis.

## Methods

### Study population

Eight male patients (mean age 56 ± 13 years) underwent CMR within 9 ± 3 days (range 5-14 <space>days) after acute ST-segment elevation myocardial infarction and in a chronic state 139 ± 50 days (range 92–210 days) after the acute event. Repeated troponin measurements were not performed. But all patients remained clinically without onset of new cardiovascular symptoms or any cardiovascular event between both CMR scans. Patients’ characteristics are described in Table 1. Note that patient two and four have the lowest release of myocardial enzymes but the lowest ejection fraction. In patient 2, this is attributable to a preexisting three-vessel disease with prior inferior infarction. In patient four, left-to-left collaterals might have compensated the cellular damage during LAD-occlusion. The results were compared to previously published T_1_- and T_2_-relaxation times in healthy controls [8].

**Table 1:** Patients’ characteristics at acute presentation

Number	1-, 2-, or 3-Vessel disease	Culprit lesion	Peak CK [U/l]	Peak Troponine T [ng/l]	LV-EF [%]	LV-EDV-I [ml/cm]	LV-M-I [g/cm]
1	2	RD	1026	1656	69	0.8	0.8
2	3	LAD	149	1122	30	1.0	0.9
3	1	LAD	1696	1280	68	1.0	0.9
4	1	LAD	46	834	38	1.2	0.9
5	2	RM	4102	9017	58	1.1	0.9
6	1	RCA	3658	3239	54	1.0	0.6
7	1	RCA	1900	3749	51	1.3	1.3
8	1	RCA	3020	5304	45	1.0	0.8

RD = diagonal branch, LAD = left anterior descending, RM = obtuse marginal branch, RCA = right coronary artery; LV-EF = left ventricular ejection fraction; LV-EDV-I = left ventricular enddiastolic volume indexed by body height; LV-M-I = left ventricular mass indexed by body height

### CMR examination

All CMR examinations were performed with a 3 T MR system (Magnetom Verio, Siemens Healthcare, Erlangen, Germany). The protocol was identical for patients and healthy controls. An integrated body RF coil was employed for RF transmission and a 32-channel cardiac RF coil for signal reception if not otherwise stated. ECG was used for cardiac gating/triggering.

#### Cine imaging

Steady-state free-precession (SSFP) 2D cine images were obtained during repeated breath-holds in three long axes and in a stack of short axes (SAX) covering the left ventricle (LV) to assess wall motion and for cardiac chamber quantification. Imaging parameters were as reported recently [8].

#### T_2_-weighted imaging

Data were acquired in basal, mid-ventricular, and apical SAX planes in end-diastole using a breath-hold, black-blood, T_2_-weighted triple inversion recovery fast-spin-echo based technique: Imaging parameters were: repetition time = 2 × R-R-interval; TE = 43 ms; inversion time for fat (TI_fat_) = 170 ms, FOV = (340×255)mm^2^, matrix = 256×192, slice thickness = 10 mm, acquisition voxel size 1.3 × 1.3 × 10 mm^3^, BW = 235Hz/px, The integrated body RF coil was used for signal transmission and reception.

#### T_2_-mapping

Data were acquired in basal, mid-ventricular, and apical SAX planes using a T_2_-prepared single-shot SSFP technique [6] as described recently [8]. Three SSFP images with different T_2_ preparation times were acquired in end-diastole within a single breath-hold. Imaging parameters were: TR = 2.4 ms, TE = 1 ms, FA = 70°, FOV = (340x278) mm^2^, matrix = 176×144, slice thickness = 6 mm, acquisition voxel size 1.9 × 1.9 × 6 mm^3^, BW = 1093Hz/px, GRAPPA acceleration factor R = 2. Images were motion corrected and a pixel-wise myocardial T_2_-map was generated. The principal accuracy of this technique has been demonstrated in previous phantom experiments [6].

#### T_1_-mapping

Data were acquired in basal, mid- ventricular, and apical SAX planes before and after administration of 0.2 mmol/kg i.v. gadobutrol (Gadovist®, Bayer Healthcare Germany). The acquisition of the post-contrast T_1_-maps was started in every examination exactly 10 minutes after gadobutrol administration, ensured by a countdown and always beginning with the basal slice. Data were obtained in end-diastole using a cardiac-gated, SSFP-based Modified Look-Locker Inversion Recovery (MOLLI) technique [7, 8]. Imaging parameters were: TR = 2.6–2.7 ms, TE = 1.0–1.1 ms, FA = 35°, FOV = (270 × 360) mm^2^, matrix = 156 × 208 to 168 × 224, slice thickness = 6 mm, acquisition voxel size 1.6–1.7 × 1.6–1.7 × 6 mm^3^, BW = 1045-1028Hz/px, GRAPPA acceleration factor 2. The hypersec adiabatic inversion pulse achieved an inversion factor of about -0.925. To generate a pixel-wise myocardial T_1_-map, single-shot SSFP images were acquired at different inversion times (pattern 3-3-5) and registered prior to a non-linear least-square curve fitting. The principal accuracy of this technique has been demonstrated in previous phantom experiments [7]. The heart rate of each subject during the MOLLI acquisition in the acute and chronic state was as follows: patient 1:59/62 beats per minute (bpm); patient 2: 83/62 bpm; patient 3: 56/55 bpm; patient 4: 51/51 bpm; patient 5: 77/65 bpm; patient 6: 62/62 bpm; patient 7: 51/50 bpm; patient 8: 57/66 bpm.

#### LGE imaging (LGE)

LGE imaging was performed 15 min after the administration of gadobutrol in the same planes as SSFP CINE imaging using a segmented inversion-recovery gradient-echo sequence. Imaging settings were as reported recently, with an acquisition voxel size 1.4 × 1.6 × 6 mm^3^ [8].

### Image analysis

Defining the myocardium within the maps was done using CMR^42^ (Circle Cardiovascular Imaging, Calgary, Canada) as previously described [8]. Much attention was invested to manually draw the endocardial and epicardial contours as accurate as possible to omit the inclusion of blood or epicardial fat.

Based on the 95 % tolerance interval of the T_2_- and T_1_-relaxation times from a previous study in healthy controls [8], thresholds that discriminate normal from abnormal T_1_ and T_2_ were defined. The cut-off for abnormal T_2_-times was >50 ms, native T_1_ > 1250 ms and post-contrast T_1_ < 350 ms. All myocardial pixels that were abnormal based on these thresholds were automatically highlighted in color in the corresponding map. The distribution of abnormal pixels was correlated with the LGE, which was regarded as the reference for the localization and extent of the infarct.

### Phantom experiments

Phantom experiments were done to evaluate the accuracy of the T_2_- and T_1_-mapping method, using the same MR scanner and coil as for the in-vivo exams. An agar-based phantom representing a range of T_1_ and T_2_ times was used. The T_1_-values were verified using an inversion recovery sequence with acquisition matrix 256x256, TR 15 s, 1 line/inversion, 90° FLASH readout, T_1_ range 200 ms–1090 ms. T_1_ values were calculated using a non-linear least square three-parameter fit. The T_2_-values were verified using a multi-echo spin echo (MESE) approach with matrix 256×256, TR 15 s, 1 segment, T_2_-range 16–235 ms. T_2_ values were calculated using a mono-exponential least square fit. For comparison, the T_2_- and T_1_-mappings technique as described for the in-vivo-measurements were applied. All phantom studies were performed with a simulated heart rate of 60 beats per minute. The signal-to-noise ratio (SNR) was estimated as the signal intensity from a manually drawn region of interest within the corresponding compartment of the phantom and the standard deviation of the signal intensity from a region of interest in the background. For T_1_, the last image of the series was used, for T_2_ the first.

## Results

### Phantom experiments

The results of the phantom experiments are shown in Table 2. They show that the applied mapping techniques provide estimates of the T_1_- and T_2_-relaxation times close to the reference technique, with MOLLI underestimating the T_1_-times. This finding is in concordance with previous studies that tested the same techniques in phantom experiments [6, 7].

**Table 2:** Relaxation times (in ms and ± SD) and estimates of the signal-to-noise ratio (SNR) of the phantom experiments

Reference T_2_	Measured T_2_	SNR estimate
39	39 ± 3	1412.4
58	65 ± 9	1360.6
83	77 ± 8	2386.9
Reference T_1_	Measured T_1_	SNR estimate
286	250 ± 14	571.4
520	473 ± 10	649.8
630	590 ± 14	478.9
925	880 ± 41	1106.5
1090	1062 ± 9	1370.5

The reference T_2_ was acquired with multi-echo spin echo, the reference T_1_ with inversion recovery. The measured T_2_ and T_1_ is based on the T_2_- and T_1_-mapping as described for the in-vivo-measurements. The estimate of the SNR stems from the first image of the T_2_-series and the last of the T_1_-series

### In-vivo-measurements

**Fig. 1:**
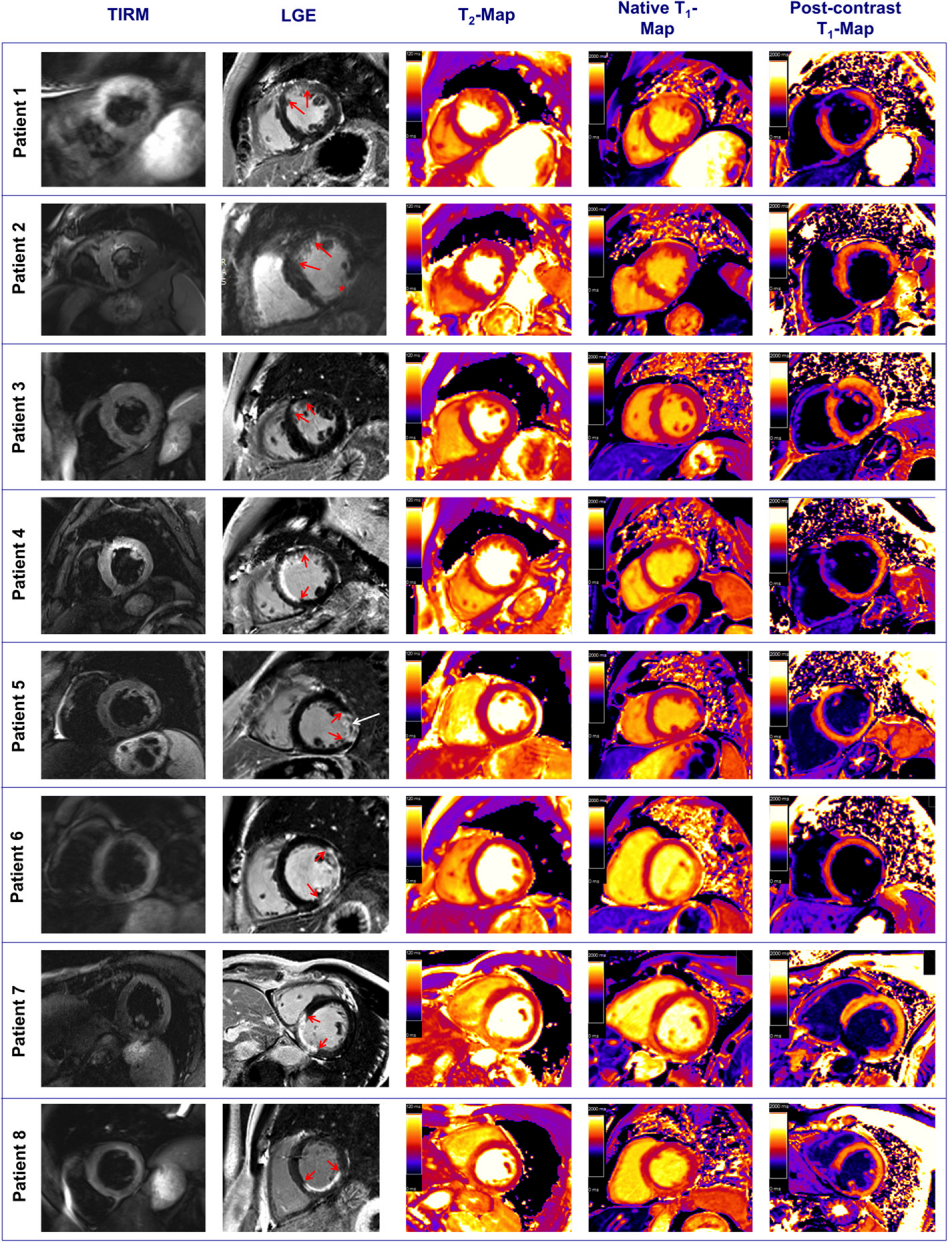
Representative images obtained with T_2_-weighted imaging, late enhancement (LGE), T_2_-map, native T_1_-map and post-contrast T_1_-map for each patient. The order of the patients corresponds to Table 1. The red arrows in the LGE images highlight the infarct region. Note that patient #2 had an old inferior infarction (red asterisk) but actually presented with LAD occlusion. Patient five had microvascular obstruction with hemorrhage (white arrow)

**Fig. 2:**
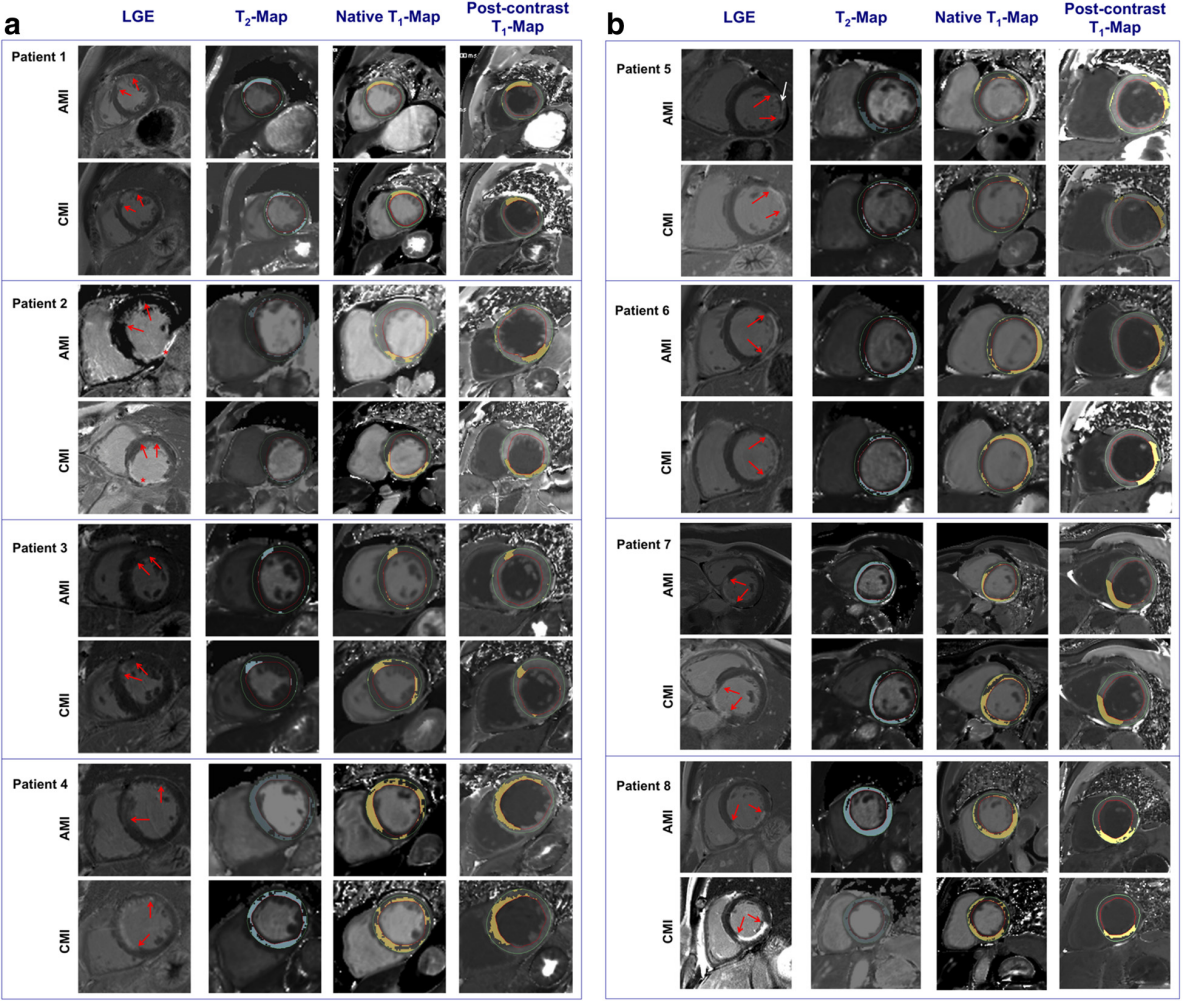
**a** and **b**. Thresholds that discriminate normal from abnormal T_1_ and T_2_ relaxation times for every myocardial pixel were defined based on reference T_2_-and T_1_-values from healthy controls. These thresholds were applied on the T_2_- and T_1_-maps so that all abnormal pixels in the myocardium (between the red and green contour) that were outside the normal range became highlighted in a color (blue in T_2_-map, brown in T_1_-maps). The red arrows in the LGE images highlight the infarct region. Note that patient #2 had an old inferior infarction (red asterisk) but actually presented with LAD occlusion. Patient 5 had microvascular obstruction with hemorrhage (white arrow). a = subjects 1-4; b = subjects 5-8

All patients had evidence of AMI using LGE and T_2_-weighted imaging during the initial scan. Figure 1 provides images of the various imaging techniques for all subjects. Note that patient #2 had an old inferior infarction (red asterisk) but actually presented with LAD occlusion. Figure 2a and b provides the T_2_- and T_1_-maps for each patient with all myocardial pixels that were beyond the predefined threshold highlighted in color. The absolute T_2_- and T_1_-relaxation times for every subject and every myocardial segment are given in Table 3.

**Table 3:** The T_2_-and T_1_-relaxation times for each patient (#1-8) and each myocardial segment (Sg. 1-16)

#	Map	Time	Sg. 1	Sg. 2	Sg. 3	Sg. 4	Sg. 5	Sg. 6	Sg. 7	Sg. 8	Sg. 9	Sg. 10	Sg. 11	Sg. 12	Sg. 13	Sg. 14	Sg. 15	Sg. 16
1	T_2_	AMI	**58.5**	49.9	46.4	49.4	45.5	43.2	**69.1**	47.4	47.0	47.3	44.8	52.9	46.0	47.7	50.5	60.9
		CMI	**48.7**	47.9	49.7	46.8	46.0	46.0	**53.4**	49.7	49.7	50.1	49.6	48.1	48.2	51.8	50.1	46.3
	Native T_1_	AMI	**1353.5**	1216.2	1168.3	1188.4	1090.2	1111.0	**1393.9**	1205.9	1177.5	1137.8	1127.8	1282.2	-	-	-	-
		CMI	**1387.8**	1223.3	1182.1	1181.1	1181.2	1106.7	**1412.7**	1180.3	1162.7	1074.9	1115.9	1274.3	1243.1	1247.6	1166.0	1232.4
	Post-cm T_1_	AMI	**290.8**	368.7	419.0	406.1	414.1	414.4	**231.2**	364.8	404.5	404.4	413.4	413.4	318.8	364.6	398.7	382.0
		CMI	**430.4**	359.8	414.1	416.1	414.1	437.8	**236.3**	369.8	366.1	444.7	391.1	326.5	346.9	366.9	416.7	417.7
2	T_2_	AMI	43.9	45.0	**42.4**	**-**	-	43.0	51.0	**55.3**	**46.4**	**50.6**	44.9	46.9	52.3	**55.3**	**49.5**	51.3
		CMI	51.2	42.8	**66.8**	**63.7**	50.1	48.4	48.8	**47.9**	**55.6**	**55.5**	42.8	43.1	46.3	**50.1**	**49.3**	44.3
	Native T_1_	AMI	1122.0	1134.3	**1280.7**	**-**	-	1076.3	1214.8	**1288.5**	**1200.6**	**1056.6**	1075.7	1158.8	1253.1	**1327.7**	**1246.3**	1140.8
		CMI	1059.3	1156.2	**1383.4**	**1364.8**	1235.9	1069.6	1059.7	**1156.2**	**1383.4**	**1364.8**	1235.9	1069.9	1196.9	**1239.7**	**1146.4**	1155.8
	Post-cm T_1_	AMI	423.6	415.0	**341.6**	**374.7**	280.4	431.8	360.2	**341.0**	**378.4**	**251.0**	423.7	398.5	351.9	**346.9**	**376.7**	406.5
		CMI	416.2	403.9	**285.3**	**289.4**	324.6	426.4	328.4	**337.4**	**382.7**	**306.1**	451.3	429.0	379.7	**365.9**	**442.9**	436.9
3	T_2_	AMI	**51.1**	**44.4**	44.9	45.2	44.6	43.3	**52.8**	**45.2**	43.8	42.4	40.3	43.7	**57.4**	**62.7**	45.9	43.9
		CMI	**49.9**	**42.1**	44.1	43.9	44.1	44.2	**49.9**	**43.2**	43.1	42.1	42.6	41.1	**45.7**	**48.4**	43.7	44.3
	Native T_1_	AMI	**1244.2**	**1144.2**	1135.5	1176.8	1119.9	1129.3	**1217.4**	**1185.6**	1174.8	1168.0	1139.0	1170.7	**1239.0**	**1444.0**	1213.3	1212.6
		CMI	**1160.0**	**1134.0**	1117.8	-	-	-	**1300.0**	**1141.5**	1152.0	1216.5	1123.4	1184.6	**1253.0**	**1250.6**	1204.9	1182.8
	Post-cm T_1_	AMI	**409.6**	**468.6**	471.8	475.7	458.6	458.4	**413.7**	**383,0**	441.7	492.3	463.4	462.9	**411.2**	**333.7**	467.2	460.3
		CMI	**403.9**	**421.8**	419.1	440.8	-	429.4	**362.4**	**422.0**	445.2	438.4	450.5	433.0	**348.0**	**349.4**	436.8	416.9
4	T_2_	AMI	**56.5**	**58.0**	**51.8**	50.5	49.6	46.5	**-**	**62.4**	**58.7**	53.5	49.6	53.5	**61.5**	**57.0**	56.9	60.2
		CMI	**51.7**	**56.9**	**61.4**	53.5	54.1	49.5	**57.0**	**53.8**	**54.8**	53.8	51.1	54.7	**53.7**	**52.7**	54.3	51.6
	Native T_1_	AMI	**1326.1**	**1344.8**	**1271.8**	1184.9	1272.8	1246.7	**1370.8**	**1334.8**	**1327.6**	1193.3	-	1215.9	**1388.5**	**1473.2**	1304.5	1155.1
		CMI	**1171.9**	**1230.0**	**1364.6**	1284.5	1249.3	1102.7	**1224.9**	**1269.1**	**1278.4**	1199.9	1186.8	1233.2	**1236.9**	**1278.9**	1241.6	1184.5
	Post-cm T_1_	AMI	**-**	**337.5**	**383.8**	394.0	444.4	418.4	**-**	**327.0**	**394.9**	423.5	417.7	379.6	**329.3**	**315.9**	390.6	412.3
		CMI	**-**	**310.1**	**362.3**	461.1	456.9	421.5	**369.3**	**310.1**	**362.3**	451.3	455.5	421.5	**372.8**	**322.7**	430.1	411.9
5	T_2_	AMI	45.7	45.0	51.5	51.2	**56.7**	**55.2**	48.2	44.5	50.3	48.9	**58.1**	**53.5**	52.7	47.0	47.2	57.2
		CMI	45.0	44.4	48.0	47.7	**47.7**	**48.4**	55.5	43.2	43.1	52.9	**47.0**	**48.5**	47.2	50.2	48.6	44.6
	Native T_1_	AMI	1124.3	1206.7	1195.1	1207.0	**1205.9**	**1128.9**	1060.3	1138.6	1169.2	1179.3	**1199.7**	**1158.1**	1098.9	1168.6	1201.4	1305.3
		CMI	-	-	1202.1	1190.3	**1225.7**	**1211.2**	1138.6	1139.2	1175.5	1368.9	**1276.1**	**1205.1**	1148.9	1148.8	-	1212.1
	Post-cm T_1_	AMI	484.1	464.7	465.3	482.4	**391.8**	**387.0**	473.4	489.3	485.8	497.0	**405.2**	**395.4**	436.8	456.8	513.0	415.3
		CMI	326.9	332.1	332.8	333.4	**275.9**	**228.8**	489.3	456.3	468.8	483.1	**379.7**	**330.0**	441.7	449.7	490.0	448.7
6	T_2_	AMI	46.3	45.2	47.5	52.4	**56.5**	**54.5**	48.2	46.6	47.1	51.0	**59.5**	**50.4**	44.6	48.4	64.7	**55.8**
		CMI	46.5	46.2	49.5	49.2	**57.7**	**51.1**	48.1	44.0	48.8	53.1	**56.8**	**48.7**	48.1	53.1	56.8	**48.7**
	Native T_1_	AMI	1169.3	1242.5	1228.4	-	**1406.4**	**1376.2**	1224.6	1230.6	1257.8	1269.2	**1350.0**	**1379.3**	1160.4	1200.0	1413.6	**1359.2**
		CMI	1356.9	1247.2	1219.7	1205.7	**1332.1**	**1430.5**	-	-	-	-	**-**	**-**	1146.1	1178.7	1264.5	**1300.7**
	Post-cm T_1_	AMI	474.0	474.0	474.2	447.9	**334.9**	**356.7**	485.1	432.3	465.8	370.5	**346.1**	**477.9**	480.0	471.8	392.2	**428.8**
		CMI	334.3	370.5	360.7	359.0	**303.6**	**246.7**	327.2	383.7	395.6	294.1	**303.1**	**379.2**	402.2	406.4	380.5	**276.5**
7	T_2_	AMI	51.7	55.4	**73.7**	**77.3**	55.3	54.5	51.1	57.2	**76.6**	**79.9**	52.0	52.6	-	-	-	-
		CMI	48.2	49.0	**48.5**	**56.8**	-	46.7	51.1	51.6	**61.9**	**55.4**	50.6	49.7	48.1	50.9	56.3	45.4
	Native T_1_	AMI	1202.6	1333.5	**1423.4**	**1539.3**	1328.1	1216.3	1120.2	1246.5	**1522.5**	**1474.8**	1214.9	1186.9	-	-	-	-
		CMI	1183.5	1234.8	**1326.9**	**1395.0**	1216.2	1248.2	1158.1	1245.3	**1334.1**	**1298.4**	-	1207.3	1198.3	1254.1	1354.8	-
	Post-cm T_1_	AMI	517.1	484.0	**329.7**	**289.8**	463.3	511.3	527.3	485.6	**342.3**	**399.0**	523.1	518.8	-	-	-	-
		CMI	480.0	463.6	**342.0**	**280.8**	436.7	471.2	492.0	462.0	**426.9**	**336.1**	-	485.7	491.9	461.9	332.9	483.2
8	T_2_	AMI	51.2	53.4	63.8	**58.3**	**57.1**	51.8	53.0	51.5	66.9	**63.9**	**58.8**	54.7	55.1	54.5	**62.4**	57.6
		CMI	50.3	53.6	56.0	**54.7**	**46.4**	49.3	47.8	49.4	59.1	**61.8**	**50.8**	47.8	48.9	50.3	**59.1**	48.6
	Native T_1_	AMI	1182.4	1196.6	1338.9	**1402.0**	**1329.1**	1194.3	1157.9	1216.7	1396.3	**1383.0**	**1268.0**	1224.3	1207.2	1279.9	**1500.1**	1296.4
		CMI	1247.9	1236.2	1299.9	**1291.7**	**1229.7**	1228.7	1184.6	1190.6	1311.3	**1350.4**	**1253.1**	1246.0	1215.2	1241.6	**1346.4**	1316.8
	Post-cm T_1_	AMI	465.5	441.5	374.6	**331.4**	**386.7**	457.2	434.6	423.8	367.5	**314.7**	**411.0**	435.6	424.4	414.8	**331.5**	429.8
		CMI	492.2	503.4	505.4	**350.5**	**386.5**	525.4	512.3	547.9	515.5	**385.6**	**551.5**	558.6	509.3	547.7	**480.2**	541.0

The myocardial segments that were affected by myocardial infarction based on the LGE images are in Bold

Only in half of the subjects with AMI, abnormal T_2_-relaxation times corresponded well with the infarcted pixels as defined by LGE [subjects 1–3, 6]. In the others [subjects 4, 5, 7, 8], pixels with T_2_-values higher than the normal range were present, but did not match with the infarcted territory as defined clinically and by LGE. In CMI (where no increase of the T_2_-relaxation time was expected), pixels with T_2_-values higher than the normal range were present in all subjects. In some of them, these pixels matched with the chronically infarcted territory [subjects 1–3, 6].

Similarly, abnormal native T_1_ times corresponded with acutely infarcted pixels in five out of eight subjects [subjects 1, 3, 6–8] in AMI, but were also present remote from the infarcted territory [subjects 4–6, 8]. In CMI, again abnormal native T_1_ values corresponded with infarcted pixels in most of the subjects [subjects 1–3, 5–8], but native T_1_ vales were also abnormal remote from the infarcted territory [subjects 3, 4, 6–8]. Pixels with abnormal post-contrast T_1_-relaxation times matched very well with LGE in all subjects both in AMI and CMI.

## Discussion

Several reports described differences of the T_1_- and T_2_-relaxation times between infarcted myocardial segments and remote myocardium [10, 11]. It seems easy to reproduce areas of myocardial infarction using T_2_- or T_1_-maps if the localization of myocardial infarction is known. However, if the investigator is blinded to any clinical information, the discrimination of normal from abnormal myocardium as well as acutely from chronically injured myocardium solely based on T_2_- and T_1_-maps is challenging, as demonstrated in the present case series.

The observed distribution of abnormal and normal relaxation times did not match closely with the extent of the myocardial lesion as defined by LGE, particularly in the T_2_-maps and the native T_1_-maps. In CMI, pixels with elevated T_2_-value that would indicate edema appeared in the myocardium. And both in AMI and CMI, pixels with abnormal T_1_- and T_2_-values appeared also in the remote myocardium, which is supposed to have values widely within the normal range.

This mismatch between the results of the thresholding and the expected distribution of T_2_- and T_1_-relaxation times within the myocardium may be attributed to several factors:

i.) A challenge of this mapping approach is the inter-individual scatter of myocardial T_2_- and T_1_-relaxation times that can be large in relation to the small difference between normal and abnormal myocardium [8]: The normal T_1_-value in healthy controls in the midventricular portion of the LV was reported to range from 1005 ms to 1296 ms at our institution and other sites report mean values of 1169 ± 73 ms mixed standard deviation [8, 13]. On the other hand, some authors reported a mean native T_1_-value of 1257 ± 97 ms for acutely infarcted segments compared to 1196 ± 56 ms for normal unaffected segments in the same subject [10]. Similarly, the normal T_2_-values in healthy controls ranged from 38 to 59 ms at 3 T and 46 to 69 ms in a study at 1.5 T [8, 14]. On the other hand, a T_2_ value of 60 ms has been reported as an adequate cutoff to determine active myocarditis [15]. Therefore, using one simple threshold based on normal values with a large scatter is probably imperfect for the individual subject. For example, in a person with T_1_-values in the lower range of the reference, the T_1_-values may still be within the normal range even after an infarct-related increase of 150 ms. This concept certainly needs further analysis in studies with larger samples. The large normal range is presumably attributable to physiological variations as well as to many other influencing factors like a potential heart-rate dependency of some acquisition methods and partial volume effects as outlined below. A detailed description of factors influencing the precision and accuracy of T_1_-measurements is available elsewhere [16]. In future, new ways of image post-processing may correct for some of these influencing factors, as recently demonstrated by Xanthis et al. [17]. An alternative approach to analyze maps - instead of defining thresholds based on the relaxation time - maybe the analysis based on the signal intensity. Kali et al. recently studied the use of native T_1_-maps in CMI. CMI was defined as using the mean ±5 standard deviation criterion relative to the respective reference regions of interest. Using this approach, native T_1_-maps and LGE images showed a close agreement to determine regions with CMI [18].
ii.) Another aspect is the influence of partial volume. Pixels that include blood or epicardial fat quickly reach pathologic T_2_- and T_1_-values and are misleadingly classified as abnormal. Even though all attempts were made to minimize this error by drawing the contours exactly within the compact myocardium, a significant influence of partial volume effects still has to be assumed. Higher spatial resolution may solve this problem in the future, and single pixels with abnormal values located at the edge of the myocardium have to be interpreted with caution.
iii.) In this case series microvascular obstruction was detected by LGE images in only one subject, therefore it does not explain the frequent mismatch between the thresholding and the expected distribution of abnormal T_2_- and T_1_-values. But generally, both T_2_- and T_1_-maps have been reported to be affected by microvascular obstruction leading to “hypoenhancement” within the “hyperenhanced” acute infarction [19]. Therefore, if large enough, microvascular obstruction may contribute to an inaccurate determination of the infarct area on T_2_- and T_1_-maps.

### Limitations

The heart rate can influence T_1_-values by affecting the relaxation between the MOLLI segments. In this study, the heart rate ranged from 50 to 83 bpm. Together with the small sample size (*n* = 8), these may be the major factors for data overlapping. Using a different mapping sequences with less heart-rate sensitivity, such as the 5(3 s)3 MOLLI variant, could have resulted in an improved performance of T_1_-mapping [16]. A limitation of the phantom experiments is that SNR has only been estimated, because no correction for multi-element coils has been performed [20, 21].

## Conclusions

This pilot study demonstrated that T_2_- and T_1_-maps with a simple threshold-based analysis did not facilitate the detection of myocardial infarction and the discrimination of AMI and CMI in the individual patient. Despite all enthusiasm for myocardial mapping, improvements in the technology as well as additional concepts for discriminating normal from abnormal myocardium may be necessary.

### Ethics approval and consent to participate

Informed consent was obtained from each patient and the study protocol conforms to the ethical guidelines of the 1975 Declaration of Helsinki as reflected in a priori approval by the institution’s human research committee (Charité Medical Faculty, EA2/077/10).

### Consent for publication

Not applicable.

### Availability of data and materials

The datasets supporting the conclusions of this article, including images, contours and databases, are stored on the institutional file server (smb://fs-cmrt.ecrc-berlin.com). The data will not be shared publicly at the current stage, as this study is part of a multi-element project that is still ongoing. Of course, data will be shared on request.

## Abbreviations

AMI: acute myocardial infarction
CMI: chronic myocardial infarction
LGE: late Gadolinium enhancement
LV: left ventricle
